# Supplementary material for: Advertising ultra-processed foods around urban and rural schools in Kenya
Source: PLOS Glob Public Health. 2025 Jun 23;5(6):e0003774. doi: 10.1371/journal.pgph.0003774 (PMC12185005; doi:10.1371/journal.pgph.0003774)
Supplement: S1 Table — (DOCX) [file pgph.0003774.s001.docx]

**SUPPORTING MATERIALS**

**S1_Table 1: NOVA food categories by different factors**

|  | **Processed Culinary Ingredient (N=30)** | **Unprocessed/ Minimally Processed (N=1132)** | **Processed Foods (N=25)** | **Ultra-processed Foods (N=1113)** | **Total (N=2300)** | **P-Value** |
| --- | --- | --- | --- | --- | --- | --- |
| **County** |  |  |  |  |  | < 0.001 |
| Baringo County | 7 (23.3%) | 52 (4.6%) | 0 (0.0%) | 107 (9.6%) | 166 (7.2%) |  |
| Nairobi County | 18 (60.0%) | 622 (54.9%) | 9 (36.0%) | 607 (54.5%) | 1256 (54.6%) |  |
| Mombasa County | 5 (16.7%) | 458 (40.5%) | 16 (64.0%) | 399 (35.8%) | 878 (38.2%) |  |
| **SES Level** |  |  |  |  |  | 0.010 |
| Low SES | 22 (73.3%) | 555 (49.0%) | 13 (52.0%) | 602 (54.1%) | 1192 (51.8%) |  |
| High SES | 8 (26.7%) | 577 (51.0%) | 12 (48.0%) | 511 (45.9%) | 1108 (48.2%) |  |
| **School setting** |  |  |  |  |  | < 0.001 |
| Urban | 26 (86.7%) | 1076 (95.1%) | 24 (96.0%) | 1012 (90.9%) | 2138 (93.0%) |  |
| Rural | 4 (13.3%) | 56 (4.9%) | 1 (4.0%) | 101 (9.1%) | 162 (7.0%) |  |
| **Gender** |  |  |  |  |  | 0.334 |
| Boys School | 1 (3.3%) | 58 (5.1%) | 1 (4.0%) | 38 (3.4%) | 98 (4.3%) |  |
| Girls School | 2 (6.7%) | 67 (5.9%) | 2 (8.0%) | 51 (4.6%) | 122 (5.3%) |  |
| Mixed School | 27 (90.0%) | 1007 (89.0%) | 22 (88.0%) | 1024 (92.0%) | 2080 (90.4%) |  |
| **School ownership** |  |  |  |  |  | 0.191 |
| Private school | 20 (66.7%) | 801 (70.8%) | 18 (72.0%) | 741 (66.6%) | 1580 (68.7%) |  |
| Public school | 10 (33.3%) | 331 (29.2%) | 7 (28.0%) | 372 (33.4%) | 720 (31.3%) |  |
| **School type** |  |  |  |  |  | 0.870 |
| Primary school | 24 (80.0%) | 904 (79.9%) | 20 (80.0%) | 873 (78.4%) | 1821 (79.2%) |  |
| Secondary school | 6 (20.0%) | 228 (20.1%) | 5 (20.0%) | 240 (21.6%) | 479 (20.8%) |  |
