# Supplementary material for: Advertising ultra-processed foods around urban and rural schools in Kenya
Source: PLOS Glob Public Health. 2025 Jun 23;5(6):e0003774. doi: 10.1371/journal.pgph.0003774 (PMC12185005; doi:10.1371/journal.pgph.0003774)
Supplement: Attachment — Submitted filename: Point-to-PointResponses-PGPH-D-24-02106_CK.docx [file pgph.0003774.s003.docx]

**RESPONSE TO COMMENTS MANUSCRIPT ID: PGPH-D-24-02106**

**Minor Comments**

Please use the space provided to explain your answers to the questions above. You may also include additional comments for the author, including concerns about dual publication, research ethics, or publication ethics. (Please upload your review as an attachment if it exceeds 20,000 characters)

**Reviewer One**

**Comment:**  The aim was to assess the extent, content, type, and power of advertising of ultra-processed/unhealthy foods around schools in both urban and rural settings in Kenya. The study presents a detailed methodology using a reference instrument applied worldwide. The results are adequately presented and discussed, with implications for public policy. I suggest adjusting minor points before publication to make the manuscript clearer.

**Response:** Thank you for the positive feedback. I will address the suggestions accordingly.

**Introduction Section**

**Comment:** The introduction is interesting and presents a scenario about advertising ultra-processed foods. However, the justification for the work could be better developed.

**Response:** Thank you for your suggestion. The justification statement has been improved and the additional information reads as follows:

“This study fills the gap of identifying differential trends of marketing of high foods in places where children gather, both in urban and rural settings to enhance contextualized recommendations. Furthermore, such findings will inform policy which focusses in LMIC urban areas, and in rural settings where industrialization is progressively taking root.”

**Materials and methods Section**

**Comment:** The study counties were selected for convenience, or did you do some random selection?

**Response:** The study counties were selected conveniently, to represent urban setting (Nairobi county), urban-coastal setting (Mombasa), and a predominantly rural setting (Baringo county).

**Comment:** Line 153: Since you've briefly explained what unprocessed and minimally processed foods are, I suggest you do the same for processed and ultra-processed foods.

**Response:** This has been edited from lines 154 to 160 and reads as follows:

“The NOVA food classification system classifies foods into i) unprocessed/minimally processed which are foods in their raw state that have undergone no industrialization processes and have no added sugars and salts ii) processed culinary ingredients which are mainly additives to foods products such as cooking oils, iii) processed foods which contains added nutrients such as salts and sugars, and iv) Ultra-processed foods(UPFs) which are highly processed foods that have undergone multiple industrialization processes and contains high levels of sugar, salts and fats ”

**Comment:** Include the software that you used to do the data analysis.

**Response: This has been added on lines 174-175 and reads as follows:**

“Stata Version 18 was used for Statistical analysis, and the significance level was set at α=0.05.”
**Results Section**

**Comment:** Include country and year in the title tables.

**Response:** This has been edited accordingly in all the tables.

**Comment:** Line 188 – 192: I needed clarification on these two sentences. First, you say that food and beverage advertisements are more common in Nairobi, but in the following sentence, you say that food and beverage products are more advertised in Mombasa. How it's written looks like the same information with different data. What data are you presenting in the first sentence that differs from the data you are presenting in the second sentence?

**Response:** Yes, it looks like the same information, the first statement explains the overall crude number of advertisements, but the second statement explains the density, explained by median (IQR), which is a better measure of the density of advertisements. I have added the word density in the second sentence to differentiate the two sentences from lines 199 to 202 and it reads as follows:

“According to the density of advertisements around schools; the food and beverage products were more advertised in Mombasa (Median=25, Interquartile range (IQR)= 12, 78) and Nairobi counties (Median=25, IQR=6, 69), and lower in Baringo county (Median=8, IQR; 5, 14) (Table 2).”

**Comment:** It would also be interesting to see the information you present in Table 3 stratified by school setting, SES level, and school type (public/private).

**Response:** That’s a good suggestion, however, Table 3 mainly wants to show the overall type and nature of the advertisements in all the counties. The stratification by by school setting, SES level, and school type (public/private) has been done for the tables indicating the food categories and the Poisson regression analysis table.

**Reviewer 2**

**General comments:** This study assessed the extent, content, type, and power of advertising of ultra-processed/unhealthy foods around schools in both urban and rural settings in Kenya. Overall, this is an interesting study that contributes to the food environment literature in LMICs. The results are as expected with urban areas possessing greater advertisements than rural areas, though there are some key nuances throughout that should be explained.

**Response:** Thank you for your comment. All the suggestions will be addressed accordingly.


**Comment:** - Grammar can be improved throughout

**Response:** Grammar has been cross-checked and improved throughout the document.

**Comment:** - L60 – “obesogenic environments” is not a standardized term. Would remove this

**Response:** This has been changed and reads as follows:

“Overweight and obesity are a growing global challenge largely driven by food environments and the consumption of unhealthy foods, as well as insufficient physical activity”

**Comment:** - Paragraphs 1 and 2 of the introductions are redundant. Can you condense?

**Response:** Thank you for your observation. However, the first paragraph provides a general overview of obesity and overweight, along with their link to nutrition-related NCDs. The second paragraph focuses on food environments, marketing, and the exposure of children and adolescents to these factors. In my opinion, the two paragraphs cover distinct yet important content, both of which I believe should be included.

**Comment:** - L75 – “HICs” is not defined previously

**Response:** This has been edited on line 76.

**Comment:** - L90-92 - The objective statement must be stronger. Why Kenya?

**Response:** The reason why Kenya is selected has been explained in the introductions section, and in the study justification. Kenya is a country in LMICs where industrialization and nutrition transition is taking place. A few countries in Africa has conducted similar studies, but little has been done to compare both rural and urban settings in LMICs.

**Comment:** - L115 – “sample size of 500” – specify schools here as it is unclear

**Response:** This has been edited accordingly.

**Comment:** - Why wasn’t a listing of schools in Kenya (and your three regions) used, versus an estimation of 500 schools? If this was indeed done, the methods should be rewritten to explain that you started with a listing of schools, stratified by primary, secondary or urban/rural, and you used a prevalence of 23% of unhealthy food advertisements as your outcome in your power calculation.

**Response:** Yes, indeed. The listing of schools was provided from the ministry of education, and the 500 schools were selected from this list. The section has been rewritten and reads as follows:

“The list of schools in Nairobi, Mombasa, and Baringo counties by location (county, subcounty), level, and type was provided by the Ministry of Education (MOE) Kenya. First, we estimated a sample size of 500, considering a prevalence of 23% of unhealthy food advertisements in deprived-resource settings, a power of 80%, and a margin of error of 0.05. We further adjusted this sample size considering a 10% nonresponse rate in all the settings. We employed a multistage stratified sampling technique in this study. The 500 schools were further randomly allocated proportionately to the number of schools by level (primary or secondary and type (private or public) in each county and sub-county using the stratified sampling method.”

**Comment:** - Tables 1 and 2 can be condensed into one

**Response:** Thank you for the suggestion. The two tables are presented separately because they provide different types of information. The first table outlines the general characteristics of the study population, specifically the 500 schools, and describes various factors and settings within these schools. This approach is similar to how we typically present participants' sociodemographic characteristics before delving into the main results of the study analysis. The second table, on the other hand, provides detailed information about the extent of advertisements around schools. Therefore, I believe keeping these tables separate will enhance clarity and facilitate easier interpretation for the readers.

**Comment:** - L194 – says you found a higher number of advertisements in private primary, but Table 2 suggests otherwise (public primary is higher)

**Response:** Yes, public primary is higher in terms of crude number of advertisements. The interpretation above is based on the median number of advertisements which gives a more specific density of advertisements around the schools.

**Comment:** - Food shops is very vague, and it’s intuitive that more food advertisements would be within/around food shops. Did you collect data on what type of food outlet (i.e., convenience store, sit-down restaurant, fast-food etc.)?

**Response:** The food shops represents kiosks and small-scale retail outlets around the defined radius around the schools. No, we did not collect data on they type of food outlet in this work package, however, we noted the advertisements outsides the food outlets such as kiosks around schools, and the painted shops.

**Comment:** - Discussion should explain nuances in the data such as Baringo country having a 28% increase in UPF advertisements, though having the fewest number of advertisements in total (median of 8 (5,15)) around schools.

**Response:** This has been added in the discussion section from lines 304-308 and reads as follows: “Additionally, we observed a significantly higher proportion of UPFs around schools in rural Baringo County compared to the other counties. Despite the lower overall number of advertisements around schools in this region, the majority of these advertisements were for UPFs. This suggests that the marketing and availability of UPFs have penetrated rural areas, where the majority of advertised products are unhealthy foods.”

**Comment:** - What about policy implications for healthy foods? Your findings reveal that both unprocessed/minimally processed and UPF were similar in % across regions. How can we promote healthy foods?

**Response: This has been addressed and added from lines 359-362 and reads as follows:**

“Furthermore, it is important to note that substantial levels of advertisements promoting healthier food options remain present in these settings. This highlights the need for regulations that strike a balance between the costs of healthier food options and the incentives for their promotion around schools."”
