# Supplementary material for: Advertising ultra-processed foods around urban and rural schools in Kenya
Source: PLOS Glob Public Health. 2025 Jun 23;5(6):e0003774. doi: 10.1371/journal.pgph.0003774 (PMC12185005; doi:10.1371/journal.pgph.0003774)
Supplement: Attachment — Submitted filename: RESPONSE TO COMMENTS_PGPH_Final.docx [file pgph.0003774.s004.docx]

**RESPONSE TO COMMENTS: MANUSCRIPT ID PGPH-D-24-02106R1**

**General Comments**

**Comment:** 1. Good methodology and use of a standard tool which is internationally accepted.

**Response:** Thank you.

**Comment:** 2. Good overall impact and highlight of the ever-growing struggle of LES, rural backgrounds, and the effect of Urbanisation.

**Response:** Thank you for the observation.

**Comment:** 3. Would suggest the use of a single standard terminology in the study paper - eg, core /non-core; healthy/ unhealthy, etc.

**Response:** This is well noted and has been implemented throughout the document.

**Comment:** 4. Please re-read the article to screen for grammatical and semantic errors.

**Response:** The grammatical errors have been checked and rectified throughout the document.

**Comment:** 5. Please avoid using terminologies such as approximate, about, most / least in the results section of the article, since these are definitive values you have obtained through your research; the same can be used in the discussion.

**Response:** This has been addressed in the results section.

**Comment:** 6. Could you structure and use bold formatting for your tables to bring out important information, like highlighting the significant p-values, etc.

**Response:** We have bolded the significant columns for Tables 4 and 5.

**Specific Comments:**

**Comment:** Line 78,79 -Various studies have been conducted in both LMICs and HICs illustrating the extent of marketing "of" health-harming food products to children and adolescents (12–18).

**Response:** This has been changed from lines 78 to 90 and reads as follows:

“A range of studies from both low- and middle-income countries (LMICs) and high-income countries (HICs) have documented the pervasive promotion of unhealthy food products directed at children and adolescents (12–18).”

**Comment:** Line 80 - Could you explain the meaning of the phrase "different socioeconomic deprivation areas" and the setting based on the reference provided?

**Response:** This indicates that the study areas encompassed varying levels of socioeconomic deprivation, determined using area-based indices of income, education, and employment, within the context of high-income countries.

**Comment:** Line 91 - Would suggest revising - "marketing of high foods".

**Response:** This has been rephrased in lines 92 to 94 and reads as follows:

“This study addresses a critical gap by identifying variations in the marketing of unhealthy foods in child-centered environments across both urban and rural settings, thereby informing more context-specific policy recommendations.”

**Comment:** Line 117, 118 - Would suggest revising - "deprived-resource" settings; "margin of error" to Alpha error set to 5%

**Response:** This has been changed from lines 118 to 120 and reads as follows:

“First, we estimated a sample size of 500 schools, considering a prevalence of 23% of unhealthy food advertisements in low socioeconomic areas (24), a power of 80%, and an Alpha error set to 5%. ”

**Comment:** Line 137 - Delete n - "merchandising) n was also recorded"

Response: This has been deleted as it reads as follows from lines 140 to 141:

“The format of advertisements (billboards, posters/banners, standing signs, painted walls and buildings, digital signs, and store merchandising) was also recorded.”

**Comment:** Line 138,139, 235 ( table): Suggest appropriate symbols to be used as of "medium (<1.3...." and "large(2.... " to "medium (> (more than symbol) 1.3" and "large (> ( more than symbol)

**Response:** This has been changed from lines 141 to 144 and reads as follows:

“The size of the advertisements was categorized as small (> A4 but < 1.3 m × 1.9 m), medium (≥ 1.3 m × 1.9 m but < 2.0 m × 2.5 m), or large (≥ 2.0 m × 2.5 m).”

**Comment:** Line 143: Please revise " premium offers were used" as it is not clear.

**Response:** This has been changed from lines 146 to 147 and reads as follows:

“Each food item advertisement was recorded, along with any associated promotional characters and premium offers, such as giveaways, discounts, or collectable items.”

**Comment:** Line 159 - The reason for additional sub-categorisation as a binary variable when an existing NOVA food classification requires explanation, and how this binary variable was arrived at based on the NOVA food classification which was used.

**Response:** This has been changed from lines 164 to 168 and reads as follows:

“This binary distinction was based on the NOVA classification, where Group IV (ultra-processed foods) was categorized as UPFs, and Groups I to III comprising unprocessed/minimally processed foods, processed culinary ingredients, and processed foods were grouped as non-UPFs. This reclassification allowed for clearer comparisons of the extent of UPF marketing relative to all other food types.”

**Comment:** Line 163 - Would suggest edit " school types and levels and level of" to school types and level with the degree or extent of urbanisation instead.

**Response:** This has been changed from lines 173 to 174 and reads as follows:

“Descriptive statistics were used to show the distribution of foods advertised by counties, SES areas, and other characteristics such as school types and level, with the degree or extent of urbanisation.”

**Comment:** Line 163: replace "assess" with "describe", as median and IQRs are not assessment tools, they are more descriptive statistics.

**Response:** This has been changed on lines 174 and 175 and reads as follows:

“We used medians and interquartile ranges to describe the extent of advertisements around schools.”

**Comment:** Line 171: Would suggest mentioning the tests used for post estimations.

**Response:** This has been added and reads as follows:

“Deviance and Pearson chi-square post-estimation tests were employed to assess the goodness-of-fit of the models.”

**Comment:** Line 206 / 207 - The Mean with a SD above the Mean may not be a great description of the dataset. Could consider Median and IQR.

**Response:** Yes, the Median (IQR) has been applied to the analysis and reads as follows:

“The Median (Interquartile Range (IQR)) school population in all the counties was 335(176, 500) pupils.”

**Comment:** Line 248/249 - It describes conversely results as described, however, based on the data, they both highlight that noncore or unhealthy foods are more advertised than the other. Also prefer standard terms - either core and noncore or healthy and unhealthy.

**Response:** Agreed, there is no contrast. The non-core foods imply the unhealthy foods advertised. This has been changed and reads as follows from lines 244 to 248:

“In Nairobi County, 44.4% of the advertisements promoted unhealthy foods (median = 37.5, IQR = 16.5–47.5), while 46.7% featured healthy foods (median = 79, IQR = 56–114) (Tables 4 and 5). In Mombasa County, healthy food advertisements accounted for 49.8% of the total, while unhealthy foods made up 44.8%. In Baringo County, unhealthy foods comprised approximately 63.9% of food and beverage advertisements (median = 15, IQR = 53–72) (Tables 4 and 5).”

**Comment:** Line 329 - Could rephrase the sentence " restricted to advertising" to "which are restricted for advertising to children in Kenya"

**Response:** This has been changed from lines 327 to 328 and reads as follows:

“Surprisingly, 3% of the advertisements were for alcohol, which is restricted from advertising to children in Kenya.”

**Comment:** Line 355: It is mentioned that "different geographical contexts" are illustrated, however, the study is more about socioeconomic stratification than geography.

**Response:** This has been changed on lines 351 and 352 and reads as follows:

“This study examines the marketing of ultra-processed foods (UPFs) in the vicinity of schools across diverse socioeconomic settings in Kenya.”

**Comment:** Line 357 - Would suggest "Better pester power to caregivers to 'pester power of children to their caregivers"

**Response:** This has been changed from lines 352 to 354 and reads as follows:

“The promotion and marketing of unhealthy foods can influence the perception of the product and thus increase the purchase intention of children or better pester power to caregivers.”

**Comment:** Line 359 - Expand NR-NCD.

**Response:** Expanded on lines 354 to 356 and reads as follows:

“Consequently, this leads to the consumption of unhealthy foods associated with poor health outcomes such as overweight/obesity, and nutrition-related non-communicable disease among children and adults.”
